# Nuclear export modulates TDP-43 phase transition and cytoplasmic aggregation

**DOI:** 10.64898/2025.12.16.694670

**Published:** 2026-04-12

**Authors:** Natalie Chin, Qi Zhang, Jizhong Zou, Ken Chih-Chien Cheng, Wei Zheng, Yihong Ye

**Affiliations:** 1 Laboratory of Molecular Biology, National Institute of Diabetes, Digestive, and Kidney Diseases, National Institutes of Health, Bethesda, MD, 20892, USA; 2 Therapeutic Development Branch, National Center for Advancing Translational Sciences, National Institutes of Health, Bethesda, MD, 20892, USA; 3 iPSC Core, National Heart, Lung, and Blood Institute, National Institutes of Health, Bethesda, MD, 20892, USA; 4 Functional Genomics Laboratory, National Center for Advancing Translational Sciences, National Institutes of Health, Bethesda, MD, 20892, USA; 5 Current address: Chinese Institute for Brain Research, Peking Union Medical College and Chinese Academy of Medical Sciences, Beijing, China

**Keywords:** TDP-43, anisosome, protein aggregation, nuclear transport, XPO1, liquid-liquid phase separation/LLPS, Amyotrophic Lateral Sclerosis (ALS), Frontotemporal Dementia (FTD)

## Abstract

RNA-binding protein TAR DNA-binding protein 43 (TDP-43) can form liquid-like, nuclear assemblies whose phase behavior may influence its aggregation propensity and neurotoxic activity. The mechanism(s) that modulates the transition of TDP-43 from a liquid to solid phase is poorly defined. Here we combine chemical and genome-wide genetic screenings to identify cellular factors that modulate the phase behavior of an RNA-binding defective TDP-43 mutant that mimics an Amyotrophic Lateral Sclerosis (ALS)-associated variant. Our screens uncover multiple cellular processes including RNA splicing, protein translation, proteostasis imbalance and nuclear export as TDP-43 phase regulators. Importantly, TDP-43 phase transition can be dynamically recapitulated in vitro in a semi-permeabilized cell system, which reveals that the inhibition of nuclear export reshapes the nuclear environment in favor of an RNA-dependent TDP-43 liquid-liquid phase separation (LLPS) state, which mitigates cytoplasmic TDP-43 aggregation. We validated this mechanism in a brain organoid model bearing an ALS-associated mutation, showing that nuclear export deficiency can limit pathogenic phospho-TDP-43 accumulation. These findings establish nuclear export as a key regulator of TDP-43 phase transitions and define a mechanistic framework that links altered nuclear transport and phase dynamics to TDP-43 aggregation potential.

The RNA-binding protein TAR DNA-binding protein 43 (TDP-43), encoded by the *TARDBP* gene, plays a central role in RNA transport, splicing and metabolism ^1, 2^, and is the major pathological component of cytoplasmic inclusions in amyotrophic lateral sclerosis (ALS) and frontotemporal dementia (FTD) ^3–5^. TDP-43 contains two RNA-recognition motifs (RRMs), a bipartite nuclear-localization sequence, and a glycine-rich C-terminal low-complexity domain (LCD) that is intrinsically prone to aggregation ^6^. Under physiological conditions, TDP-43 binds RNAs in the nucleus, participating in multiple RNA-metabolic processes. In contrast, disease-causing mutations trigger its mis-localization to the cytoplasm, forming detergent-resistant inclusions. TDP-43-positive protein aggregates were present in ~97% of ALS and ~45% of FTD cases, as well as in a subset of Alzheimer’s disease patients. These diseases are now collectively termed TDP-43 proteinopathies ^4, 7^. Both loss of nuclear function and gain of cytoplasmic toxicity have been implicated in neurodegeneration ^2, 6^, underscoring the importance of nucleocytoplasmic transport in regulating TDP-43 patho-physiological functions.

Like many RNA-binding proteins (RBPs), TDP-43 undergoes liquid-liquid phase separation to form membraneless biomolecular condensates ^6, 8, 9^. Under conditions of cellular stress or in the presence of RNA-binding inhibiting mutations, these condensates can transition from dynamic liquid droplets to rigid gel-like or solid assemblies ^8, 10, 11^. To date, more than 70 disease-linked mutations in *TARDBP* have been identified in ALS or FTD, many within the LCD, while others clustering near or in the RRMs to disrupt RNA binding ^6^. Defects in RNA binding appears to be a major determinant of TDP-43 phase behavior ^12, 13^. Disease-associated mutations diminish TDP-43’s activities in RNA metabolism, causing widespread cryptic exon inclusion ^14–17^, alternative polyadenylation and other RNA maturation defects that collectively contribute to neurotoxicity ^18, 19^.

Apart from disease-associated mutations, post-translational modifications (PTM), especially acetylation, have been shown to modulate TDP-43’s RNA binding capacity ^16, 20^. Acetylation was detected on endogenous TDP-43 in ALS patient samples. In vitro, TDP-43 acetylation is enhanced when cells are exposed to oxidative or proteotoxic stress ^20, 21^. Interestingly, like disease-associated TDP-43 mutants, acetylated TDP-43 is also more aggregation-prone and displays a similar phase regulation pattern ^20^. For example, a recent study showed that a TDP-43 acetylation mimetic mutant designated as 2KQ and several RNA-binding defective TDP-43 disease variants can all undertake a unique form of demixing, forming “anisosomes” that contain an anisotropic spherical shell of TDP-43 and a central liquid core enriched in the HSP70 chaperone ^22^. Anisosomes, observed mostly in the nucleus under overexpression conditions, have not been validated in human patient samples. Nevertheless, they might be an intermediate en route to pathological aggregation when cellular proteostasis or nucleocytoplasmic transport is compromised, as suggested by a recent study ^22^. Additional in vitro studies using purified TDP-43 and small molecules have begun to elucidate the molecular basis of TDP-43 self-association ^23, 24^, offering insights into its phase behavior and aggregation mechanisms.

Despite these advances, the cellular pathways modulating TDP-43 phase behavior (droplet formation, anisosomal shell–core architecture, conversion to gel/solid structures) and its link to disease-associated aggregation remain unclear. We address this knowledge gap by combining a chemical-genetic screen approach with a genome-wide siRNA screen to identify molecular determinants that modulate the phase behavior of the RNA-binding defective TDP-43 2KQ mutant. Our study reveals critical contributions of RNA splicing, protein translation, and the HSP90/ubiquitin–proteasome proteostasis network to TDP-43 phase transition. Importantly, our work demonstrates how nuclear export can influence the transition of TDP-43 from a nuclear liquid-demixed form to a cytoplasmic immobile gellike structure akin to protein aggregation. Using an iPSC-derived 3-D organoid model bearing an ALS- associated mutation ^16^, we further validated the impact of nuclear export inhibition on the accumulation of cytoplasmic phosphorylated-TDP-43. Collectively, these findings establish a mechanistic framework linking altered phase dynamics and TDP-43 aggregation to nuclear transport defects, a process known to modulate neurodegeneration in ALS and FTD.

## Results

### A chemical genetic screen identified modulators of TDP-43 phase behavior

**Figure 1:**
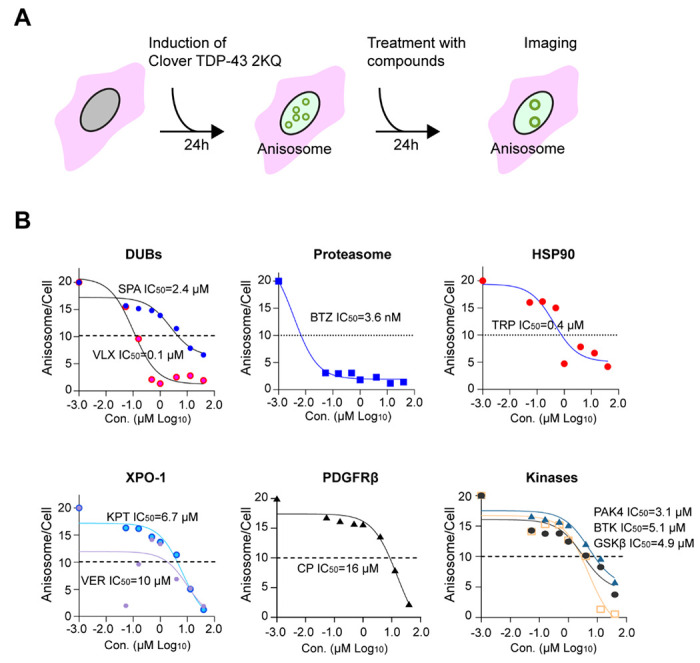
A chemical genetic screen identifies TDP-43 phase modulators **(A)** Workflow of the chemical genetic screen. **(B)** Dose dependent reduction of anisosome number by identified chemicals. DLD1 TDP-43 2KQ-Clover cells were treated with doxycycline to induce anisosome for 24 h and then treated with the drugs as indicated for 15 h. Cells were imaged for anisosome count.

To identify cellular pathways modulating TDP-43 phase behavior, we conducted a chemical genetic screen using a small molecule library targeting diverse known cellular pathways and processes. We employed a previously established DLD1 cell model stably expressing an RNA-binding defective TDP-43 mutant (2KQ) tagged with the green fluorescence protein Clover. In this system, TDP-43 2KQ undergoes rapid demixing upon induced expression ^22^, generating 20-30 sphere objects named anisosome in each nucleus. Both the number and size of anisosome increase substantially in the initial phase of TDP-43 induction but remain largely unchanged after 24 h of induction. We seeded cells in 384-well plates and induced the expression of TDP-43 2KQ with doxycycline for 24 hours. We then treated these cells with a LOPAC library containing 1280 drugs for 24 hours (Figure 1A). This approach could avoid false positive drugs that affect the expression of TDP-43 2KQ.

High content imaging combined with algorithm-based image analyses (see methods) detected ~20 spherical anisosomes per cell in controls and most treated cells. However, in cells treated with a subset of drugs, the number of anisosomes was noticeably reduced (Supplemental Table 1). Importantly, cells treated with Bortezomib, a potent proteasome inhibitor, had fewer but enlarged TDP-43 positive puncta with irregular shapes (Figure 1B) similarly to cells treated with another proteasome inhibitor as reported previously ^22, 25^. This result validated our screening design.

To further confirm the identified hits, we performed a concentration titration experiment, which showed that these inhibitors dose-dependently reduced the anisosome number with IC50 ranging from submicromolar to micromolar (Figure 1B, Supplemental Table 1). Immunoblotting showed that treatment with these drugs did not change the total TCP-43 protein level (Supplementary Figure 1), suggesting that the impact on anisosome is not due to altered protein expression. Interestingly, the screen identified cytoplasmic signaling kinases including PAK4, BTK, and GSK-3β as well as the receptor tyrosine kinase PDGFRβ as modulators of TDP-43 phase behavior (Figure 1B, Supplemental Table 1). PAK4 and GSK-3β are known regulators of the YAP signaling pathway ^26^, and YAP was recently identified as a TDP-43-interacting protein that influences its phase dynamics ^27^. These connections provide additional physiological support for the relevance of the uncovered modulators. Our screen also linked TDP-43 2KQ demixing to protein translation because cycloheximide, a ribosome elongation inhibitor also affect TDP- 43 phase dynamics (see below).

### Live cell imaging revealed two distinct classes of TDP-43 phase modulators

**Figure 2:**
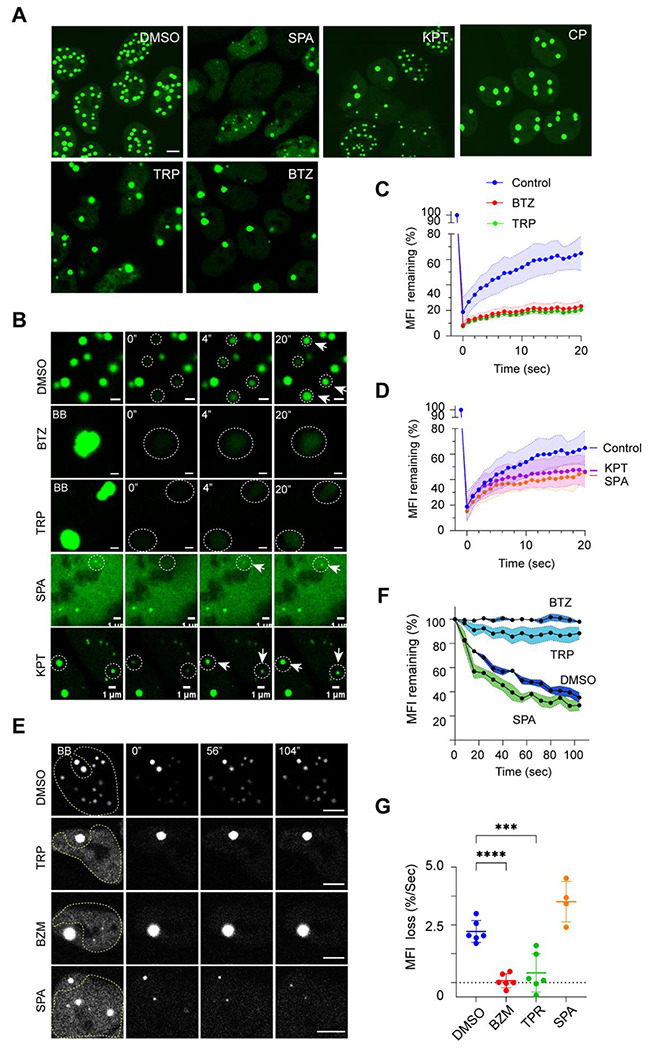
Two distinct types of TDP-43 phase modulators **(A)** DLD1 TDP-43 2KQ-Clover cells treated with doxycycline for 24 h were further treated with the indicated compounds for 6 h and imaged (SPA, Spautin-1, 5 μM; KPT, KPT-276, 15 μM; CP, CP-673451, 30 μM; TRP, Tripterin, 1 μM; BTZ, Bortezomib, 10 nM). Shown are single confocal z-section views. Scale bar, 5 μm. **(B)** FRAP analyses of anisosomes after treatment with the indicated drugs for 3-5 h. Circles indicate bleached areas. Shown are single confocal z-section views. Scale bars, 1 μm. **(C, D)** Quantification of the experiments represented in B. MFI, Mean Fluorescence Intensity. N = 5 anisosomes/condition. **(E)** Reverse FRAP analyses of anisosome dynamics. The areas indicated by the dashed lines were bleached. Shown are single confocal z-section views. Scale bar, 5 μm. **(F)** Quantification of the Mean Fluorescence Intensity (MFI) loss over time in unbleached anisosomes as shown in E. N = 7 anisosomes/condition. **(G)** Quantification of the initial rate of fluorescence loss in unbleached anisosomes as shown in E. ****, p<0.0001; ***, p<0.001 by unpaired Student’s t-test (two-tailed). N = 7 anisosomes/condition.

We next used confocal microscopy to further characterize a subset of the identified inhibitors. We chose Tripterin (a drug targeting the heat shock protein HSP90), CP-673451 (a PDGFRβ inhibitor), two deubiquitinase inhibitors VLX-1570 and Spautin-1, and the Exportin-1 inhibitor KPT-276 because previous reports have linked the targets of these compounds to TDP-43 proteinopathies ^28–31^. We first induced anisosome formation for 24 hours and then treated cells with the inhibitors. Compared to DMSO-treated controls, cells treated with Spautin-1 had fewer and smaller anisosomes. KPT-276 treatment also reduced anisosome number, albeit to a lesser extent (Figure 2A, top panels). In contrast, cells treated with CP-673451, Tripterin, or Bortezomib had fewer but larger TDP-43 punctae. Since Tripterin was also reported as a 20S proteasome inhibitor, we confirmed the role of HSP90 in this process using the well-established HSP90 inhibitor Geldanamycin (Supplemental Figure 2A). These results suggest that TDP-43 phase dynamics are modulated by the cellular folding capacity, the ubiquitin proteasome system, and nucleocytoplasmic transport.

To further dissect the effects of these inhibitors on TDP-43 phase separation, we combined drug treatment with live cell fluorescence imaging, using Fluorescence Recovery After Photobleaching (FRAP) or reversed FRAP. In FRAP, when the green fluorescence of anisosomes was bleached by a laser in untreated cells, it rapidly recovered due to the recruitment of TDP-43 2KQ from the nucleoplasm (Figure 2B, top panels, Figure 2C, D). Conversely, in reversed FRAP, bleaching TDP-43 2KQ surrounding an anisosome repeatedly over time caused a gradual decline in fluorescence intensity within the unbleached anisosome, while the surrounding areas regained fluorescence partially (Figure 2E, top panel). This result suggests that TDP-43 2KQ in anisosomes undergoes rapid exchange with a pool in the nucleoplasm. Likewise, in cells treated with Spautin-1 or KPT-276, anisosome-associated TDP-43 2KQ could freely exchange with the nucleoplasmic pool (Figure 2B, D). In contrast, in cells treated with Bortezomib, Tripterin or Geldanamycin, the enlarged TDP-43 puncta appeared static; TDP-43 2KQ fluorescence within anisosomes failed to recover after photobleaching (Figure 2B, C, Supplemental Figure 2B), nor did it decrease over time when surrounding TDP-43 2KQ was bleached (Figure 2E–G). Collectively, these results suggest that these inhibitors modulate anisosomes via two distinct mechanisms: one maintains TDP-43 in a demixed liquid state, while the other converts it to a gel-like solid state.

### A genome-wide siRNA screen identified genetic modulators of TDP-43 phase separation

**Figure 3:**
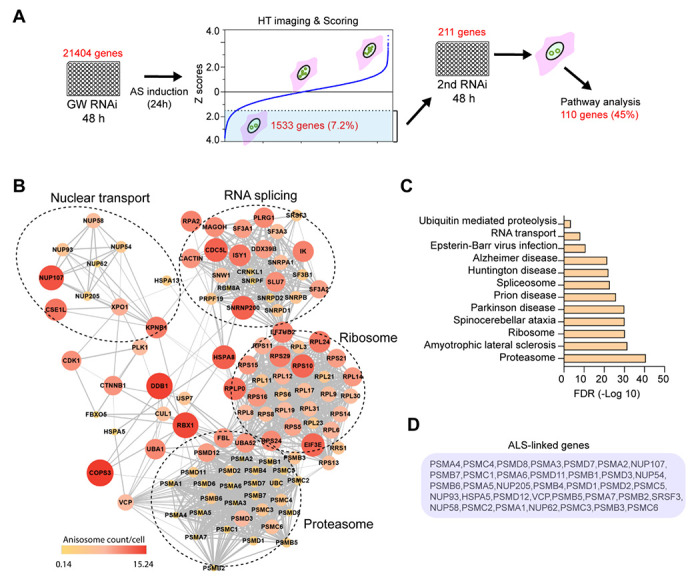
A genome-wide siRNA screen identifies modifiers of TDP43 phase behavior **(A)** The workflow of the siRNA genetic screen. GW, genome-wide; AS, anisosome; HT, high-throughput. **(B)** Pathway analysis of genes whose knockdown reduces anisosome number in cells. The relative anisosome count are indicated by both color and size of the nodes. **(C)** GO molecular function analysis of TDP-43 phase modifiers. **(D)** A list of identified genes linked to ALS in C.

To further decipher the molecular determinants of TDP-43 phase separation, we conducted an unbiased genome-wide siRNA knockdown (KD) screen (Figure 3A). To this end, we transfected a siRNA library targeting 21,404 human genes each with three siRNAs into DLD1 cells stably expressing Clover-tagged TDP-43 2KQ. After gene KD, anisosomes were induced for 24 hours. High content imaging and automated analysis identified 1,533 candidates whose KD reduced the anisosome number per cell (Z-score > 2) (Supplemental Table S2). To further narrow down the list, we performed a STRING protein network analysis based on the assumption that a protein interaction network bearing multiple positive hits would be more likely to be a true effector. We selected 211 networked genes with top Z-scores and rescreened each of them with three additional siRNAs. This suggested the involvement of 110 genes in TDP-43 anisosome regulation (Figure 3A, Supplementary Table S3). GO pathway analyses categorized these genes into several pathways including RNA splicing, protein translation, proteasomal degradation, and nuclear transport (Figure 3B). The identification of ribosomal proteins and proteasome subunits is consistent with our chemical genetic screen, which implicates translation and proteasomal degradation in anisosome modulation (Supplemental Table 1). Interestingly, GO analyses using ‘molecular function’ linked many identified genes to neurodegenerative diseases, particularly ALS (Figure 3C, D).

### Anisosome dynamics is modulated by RNA splicing and protein translation

Given the well-established function of TDP-43 in RNA binding and processing, we investigated the role of RNA splicing in anisosome regulation. To this end, we first incubated TDP-43 2KQ cells with doxycycline to induce anisosomes and then treated cells with a potent RNA splicing inhibitor Pladienolide-B (PlaB). Confocal microscopy confirmed that RNA splicing inhibition resulted in fewer but larger TDP-43-positive puncta in a dose dependent manner (Figure 4A, B, Supplemental Figure 3A). Despite size difference, anisosomes in drug-treated cells were morphologically indistinguishable from controls. FRAP experiments further showed that TDP-43 within PlaB-treated condensates remained highly mobile, although the fluorescence recovery rate was slightly reduced compared to controls (Figure 4C, D). Time-lapse live cell imaging frequently detected fusion of TDP-43 puncta after PlaB treatment (Figure 4E, Movie S1). Together, these results suggest that disrupting RNA splicing stabilizes TDP-43 in a demixed liquid state, resulting in larger condensates.

Next, we explored the effect of translation inhibition on anisosome dynamics using Cycloheximide (CHX) and Anisomycin (ANS), two translation elongation inhibitors. Confocal imaging and immunoblotting showed that translation inhibition by CHX or ANS did not significantly reduce TDP-43 expression (Supplemental Figure 1, 3). Like PlaB-treated cells, CHX- and ANS-treated cells contained fewer, enlarged anisosomes (Figure 4F–H, Supplemental Figure S2B). FRAP analysis revealed that TDP-43 dynamics within anisosomes were similar between ANS-treated and control cells (Figure 4I, J). These results suggest that translation inhibition also stabilizes TDP-43 in liquid condensates.

### Nuclear export modulates TDP-43 liquid-to-solid phase transition

Our screen also identified several nuclear transport regulators (e.g., Exportin-1/XPO1, Exportin-2/CSE1L) and nuclear pore components as anisosome regulators (Figure 3B). We focused on XPO1 because our chemical genetic screen identified two XPO1 inhibitors, KPT-276 and Verdinexor as potent anisosome modulators (Figure 1B).

**Figure 5:**
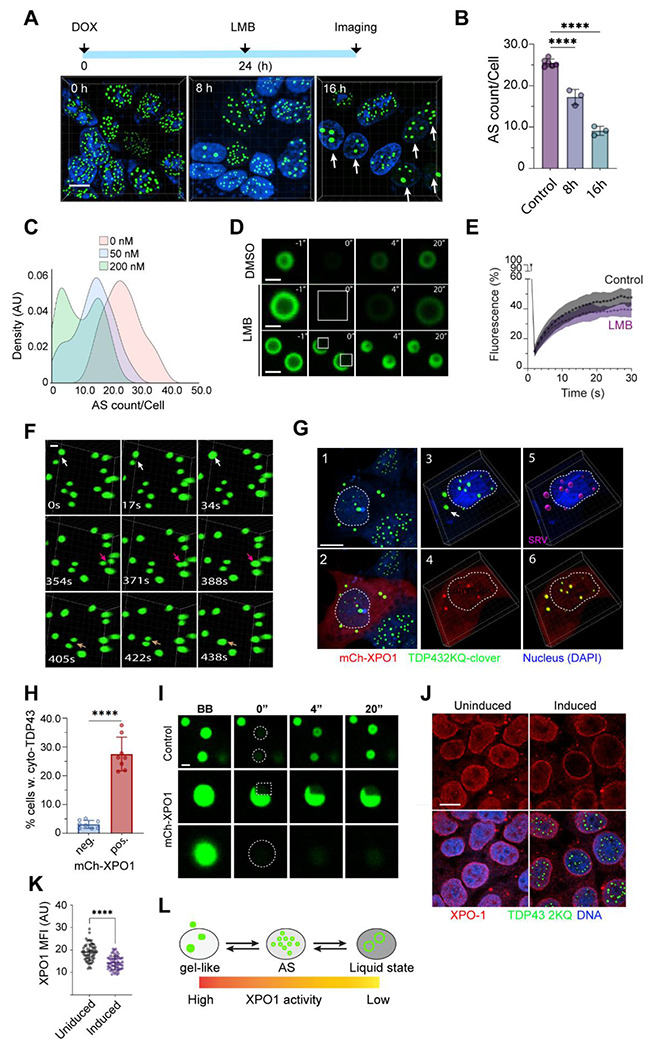
XPO1 regulates anisosome liquid-to-solid transition **(A)** Pharmacological inhibition of XPO-1 with Leptomycin B (LMB, 200 nM) reduces the number of anisosome. The graph on top indicates the experimental design. Arrows show cells with enlarged anisosomes. Scale bar 10 μm **(B)** Quantification of anisosome numbers per cell in experiments represented by A. Error bars indicate mean ± SD; **** p <0.0001 by ordinary one-way ANOVA and Dunnett’s multiple comparisons. N=3 biological repeats. **(C)** LMB dose dependently reduces anisosome number. Anisosome were induced in TDP-43 2KQ-Clover cells followed by treatment with LMB at the indicated concentrations for 16 h. The histogram shows the distribution of cells (50-90 cells/condition) by anisosome count. **(D)** FRAP experiments demonstrate that anisosomes remain in a liquid phase following LMB treatment (200 nM, 16 h). Anisosome-induced (24 h) cells were treated with DMSO or LMB for 16 h and then photobleached at the indicated areas. Scale bar 1 μm. **(E)** Quantification of the FRAP experiment in D. N=18 for control and 16 anisosomes for LMB-treated cells. **(F)** Time-lapse confocal microscopy detects anisosome fusion after LMB (200 nM, 5 h) treatment in TDP-43 2KQ-Clover cells. Arrows indicate fusion events. Scale bar, 1 μm **(G)** Overexpression of mCherry-tagged XPO-1 in TDP-43 2KQ-Clover cells induces cytoplasmic TDP-43 puncta. TDP-43 2KQ-Clover cells (green) transfected with mCherry-XPO1 (red) were stained with DAPI (blue) to label nuclei (dashed lines). Cells were imaged 48 h post-transfection. Panels 1, 2 show a representative confocal section, while panels 3-6 show reconstructed 3-D views. The position and volume of anisosomes were also presented in magenta in a surface-rendered view (SRV) in panel 5. The arrow in panel 3 indicates an example of cytoplasmic TDP-43 aggregate. Scale bar, 10 μm. **(H)** Quantification of the percentage of cells showing cytosolic TDP-43 puncta in XPO-1 positive (pos) and negative (neg) cells in randomly selected fields from 3 independent experiments. **** p <0.0001 by two-tailed unpaired Student’s t-test. **(I)** FRAP-based confocal imaging reveals the transition of anisosomes into a gel-like state upon XPO-1 overexpression. Scale bar, 1 μm. **(J)** Anisosome formation changes endogenous XPO-1 localization. TDP-43 2KQ-Clover before or after anisosome induction were stained with anti-XPO-1 antibodies (red) and DAPI (blue). The bleached areas were indicated by dashed lines. Scale bar, 10 μm. **(K)** Quantification of nuclear endogenous XPO-1 Mean Fluorescence Intensity (MPI) in individual cells (indicated by dots) before or after anisosome induction. **** p < 0.001 by two-tailed unpaired Student’s t-test. N = 58 cells for uninduced and 63 cells for induced condition in 2 independent repeats. **(L)** A schematic model depicting how the nuclear XPO-1 activity influences TDP-43 liquid-to-phase transition. AS, anisosome.

To explore the role of XPO1 in TDP-43 anisosome regulation, we induced anisosome formation in Clover-TDP-43 2KQ cells and treated them with Leptomycin B (LMB), a potent XPO1 inhibitor. LMB treatment resulted in a time- and dose-dependent reduction in anisosome number, while increasing their sizes (Figure 5A–C). The enlarged TDP-43 puncta showed the typical hollowed anisosome ring structure (Figure 5D), indicating that TDP-43 remained in liquid state. Indeed, FRAP experiments demonstrated that LMB treatment did not significantly affect the fluorescence recovery rate of TDP-43 within bleached anisosomes (Figure 5D, E). Time-lapse confocal imaging showed that shortly after LMB treatment, preformed anisosomes began to fuse with each other (Figure 5F, Movie S2). Thus, XPO1 inhibition stabilizes TDP-43 in a liquid phase.

To corroborate the inhibitor study, we overexpressed mCherry-tagged XPO1. Surprisingly, we observed fewer and enlarged TDP-43 positive puncta in mCherry-XPO1-expressing cells (Figure 5G, panels 1, 2). At first glance, this phenotype appeared similar to that of XPO1-inhibited cells. However, careful examination revealed several distinct features. First, despite the size increase, we did not observe the typical hollowed anisosome ring structure. Instead, these large puncta appeared more irregular in shape. Secondly, while anisosomes in mCherry-XPO1 negative cells were almost exclusively nuclear, ~30% of mCherry-XPO1-expressing cells contained cytoplasm-localized TDP-43 puncta (Figure 5G, panels 1, 2, 3, Figure 5H). Thirdly, FRAP experiments demonstrated that Clover-TDP-43 fluorescence did not recover after photobleaching, suggesting that TDP-43 was in a gel-like state in transition to aggregates (Figure 5I). Notably, mCherry-XPO1 could be detected within TDP-43 puncta in some cells (Figure 5G, panels 3-6). These findings are consistent with a previous report that showed the regulation of TDP-43 nuclear egress by multiple nuclear export receptors including XPO1 ^31^. As expected, overexpression of mCherry did not alter the subcellular localization of TDP-43 puncta, nor did mCherry colocalize with TDP-43 (Supplemental Figure S4).

Immunostaining of endogenous XPO1 in anisosome-induced cells showed that anisosome induction depleted XPO1 from the nucleoplasm (Figure 5J, K), further suggesting a possible connection between XPO-1 and TDP-43. Since XPO1 does not bind TDP-43 directly ^32^, an indirect interaction between these molecules might cause the depletion of XPO-1 from nucleoplasm in anisosome-induced cells. However, since antibody staining could not conclusively demonstrate the sequestration of endogenous XPO-1 in anisosomes due to an antibody penetration barrier ^22^, alternative interpretation cannot be excluded. Collectively, our results suggest that the stability and dynamics of anisosomes are modulated by XPO1-mediated nuclear export: reduced XPO1 activity stabilizes a liquid state and favors larger TDP-43 condensates, whereas increasing XPO1 activity promotes its transition to a gel-like structure (Figure 5J).

### Nuclear export inhibition stabilizes TDP-43 anisosomes in an RNA dependent manner

**Figure 6:**
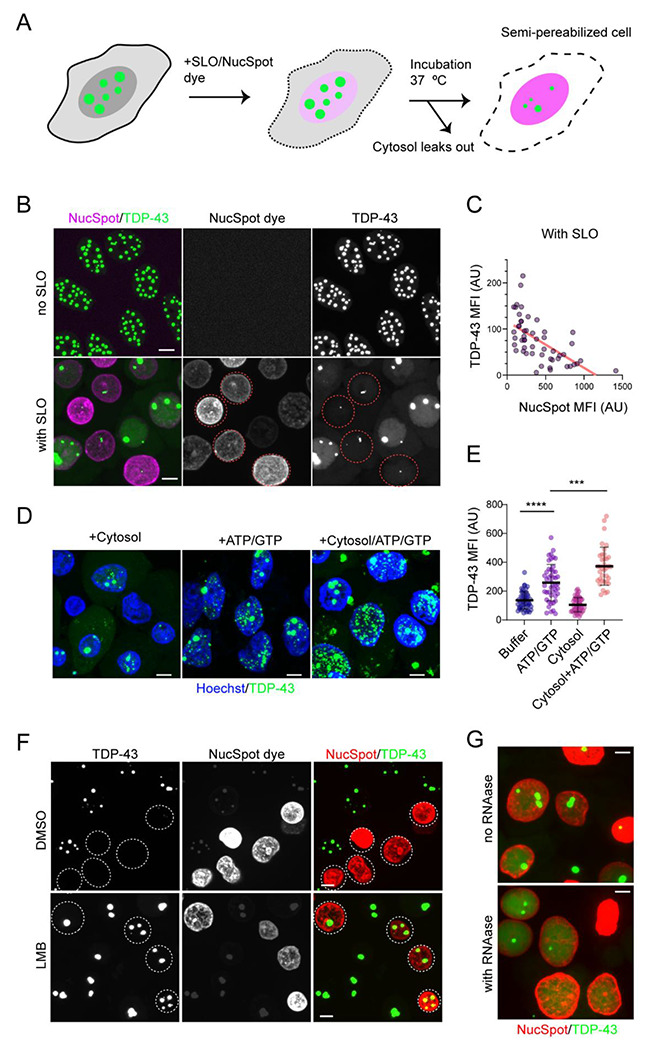
The inhibition of XPO-1 stabilizes anisosomes in an RNA-dependent manner. (A) A schematic diagram of the semi-permeabilized cell assay. SLO, streptolysin O. (B) TDP-43 in anisosomes becomes soluble after cell permeabilization (indicated by NucSpot positive staining in magenta). Note: The intensity of NucSpot dye indicates permeabilization time. Dashed circles highlight cells permeabilized early during the incubation. The cells have fewer anisosomes. Scale bars, 5 μm. (C) TDP-43 mean fluorescence intensity (MPI) displays an inverse correlation with the NucSpot dye intensity in B. AU, arbitrary unit. (D) Small TDP-43 positive puncta were reformed after permeabilized DLD1 cells were incubated with cow liver cytosol in the presence of an ATP regeneration system and GTP (100 μM) (ATP/GTP). Cells were fixed and stained with Hoechst (blue) to label nuclei. Scale bars, 5 μm. (E) Quantification of the TDP-43 Mean Fluorescence Intensity (MPI) in cells as shown in D. Error bars, SD. ****, p<0.0001; ***, p<0.001 by unpaired Student’s t-test. N=at least 35 randomly selected cells representing two independent experiments. (F) LMB treatment (200 nM, 16 h) stabilizes anisosomes. Dashed circles indicate permeabilized cells. Scale bars, 5 μm. (G) RNAase T1 treatment destabilizes anisosomes in DKD1 cells pre-treated with LMB (200 nM, 16 h). DLD1 cells induced for TDP-43 expression were treated with LMB (200nM, 16 h). Cells were permeabilized in the absence or presence of RNAase T1 for 40 min before imaging. Images shown in this figure are maximum intensity projection views of confocal z-sections. Scale bars, 5 μm.

To further dissect the role of nuclear export in TDP-43 phase regulation, we developed a semipermeabilized cell–based *in vitro* assay (Figure 6A). To this end, anisosomes were first induced in Clover-tagged TDP-43 2KQ cells, followed by selective permeabilization of the plasma membrane using the pore-forming toxin streptolysin O (SLO). Permeabilization was monitored at 37 °C by confocal microscopy using the membrane-impermeable dye NucSpot, which stained nuclei only upon plasma membrane disruption.

Notably, permeabilization led to a marked reduction in TDP-43–positive nuclear puncta and overall fluorescence intensity (Figure 6B). Quantitative analysis revealed an inverse correlation between NucSpot signal and TDP-43 fluorescence (Figure 6C). The reduction in TDP-43 signal was not due to protein elimination. Instead, TDP-43 appeared to shift from a phase-separated state to a soluble state, likely because of cytosolic factor loss and ATP depletion during cell permeabilization. Consistent with this interpretation, supplementation of permeabilized cells with cow liver cytosol together with an ATP-regenerating system (ARS) and GTP restored TDP-43 puncta (Figure 6D), albeit much smaller than anisosomes. Nevertheless, this led to a significantly increase in fluorescence intensity (Figure 6E).

The re-formed puncta may represent intermediates en route to anisosome assembly. However, these structures failed to fuse into larger anisosomes even after prolonged incubation, suggesting that cytosol add-back only partially restores the intracellular environment required for full anisosome maturation. Addition of buffer supplemented with ARS and GTP also modestly induced the formation of small TDP-43 puncta and increased fluorescence intensity (Figure 6D, E). In contrast, buffer or cytosol alone was insufficient to support puncta formation. Together, these results establish this semi-permeabilized system as a platform in which TDP-43 condensates can be dissolved and partially reconstituted under controlled conditions.

We next applied this assay to examine the mechanism underlying leptomycin B (LMB)-induced enlargement of anisosomes. Strikingly, anisosomes in LMB-treated cells remained stable following permeabilization (Figure 6F). However, inclusion of RNase T1, which degrades single-stranded RNA, during permeabilization led to their dissolution (Figure 6G). These findings suggest that LMB stabilizes anisosomes, at least in part, by increasing nuclear RNA availability.

### Nuclear export inhibition mitigates TDP-43 hyperphosphorylation in an organoid model of TDP-43 proteinopathy

Since increasing the XPO1 activity causes more cytoplasmic, gel-like TDP-43, we postulate that reducing XPO1 activity might mitigate TDP-43 cytosolic aggregation. To test this idea, we used a recently established anisosome-bearing organoid model of TDP-43 proteinopathies ^16^, which is derived from an induced pluripotent stem cell (iPSC) line carrying the ALS-associated mutation (K181E) in the endogenous TDP-43 locus ^16^. Like the 2KQ mutant, the K181E substitution disrupts the RNA binding activity of TDP-43 ^33^. Additionally, the K181E mutant was reported to accumulate in a liquid-condensed phase in association with HSP70 in cultured cells under overexpression conditions, and like anisosomes ^22^, these condensates can be converted to solid aggregates during cellular stress or HSP70 depletion ^34^.

**Figure 7:**
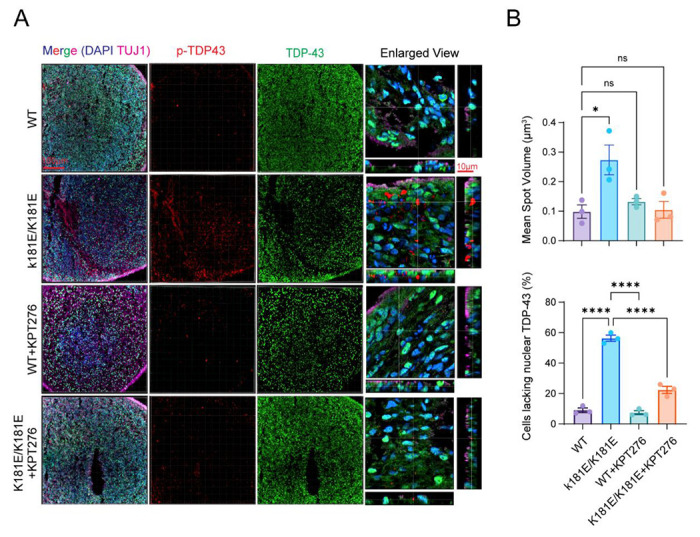
Inhibition of XPO-1 mitigates TDP-43 hyperphosphorylation in TDP-43 K181E organoids **(A)** Confocal fluorescence imaging reveals significant reduction in phosphorylated TDP-43 (p-TDP43) in K181E/K181E organoids following KPT-276 treatment. Organoids of the indicated genotypes at day 87 were treated with DMSO as a control, or with KPT-276 (20 nM) for 35 days. Organoids were fixed, sectioned, and stained with antibodies against TUJ1, a neuronal marker (Magenta), TDP-43 (green) and p-TDP-43 (red). n= 3-5 organoids from two individual batches. **(B)** Quantification of p-TDP-43 Mean Spot Volume (top) and percentage of cells lacking nuclear TDP-43 (bottom) in experiments represented by **A**. Error bars indicate mean ± s.e.m. * p < 0.05, **** p < 0.0001 by one-way ANOVA, n=3 organoids/each.

We treated organoids bearing the endogenous K181E mutation or wild-type (WT) controls with KPT-276 at a low dose (20 nM) for 35 days starting on day 87 based on pilot data indicating good tolerance with prolonged treatment. We then sectioned and stained these organoids with antibodies against Serine 409/410-phosphorylated TDP-43 (p-TDP-43) and total TDP-43 since hyperphosphorylated TDP-43 (S409/410) is a clinically validated pathological marker for TDP-43-containing cytoplasmic aggregates. In this experiment, homozygous organoids were chosen to enhance the detection of endogenous p-TDP-43. Confocal imaging revealed that WT and K181E organoids had similar amounts of total TDP-43, but p-TDP-43 was only significantly present in mutant organoids (Figure 7A), as reported ^16^. KPT-276 did not alter the total TDP-43 levels in either genotype (Figure 7A) but significantly reduced the p-TDP-43 positive puncta in K181E organoids (Figure 7A, B). This result suggests that nuclear export is required for the formation of p-TDP-43-containing aggregates in a disease relevant organoid model.

## Discussion

ALS- and FTD-associated TDP-43 mutants defective in RNA binding are known to undergo selfassociation through their intrinsically disordered regions and RNA-binding domains, generating demixed liquid, gel, and aggregated states that remain in dynamic exchange. This protein phase behavior is shared by many RNA binding proteins and is increasingly recognized as a contributor to protein aggregation in neurodegenerative diseases ^9^. Intriguingly, TDP-43 phase separation forms unique layered nuclear structures, with an HSP70-filled central core surrounded by a protein shell made of TDP-43. Although it is unclear how these structures are nucleated in the cell, our observations suggest that, once formed, they can grow by recruiting additional TDP-43 from the surrounding environment. Small anisosomes may also fuse with one another, although such fusion events become infrequent and difficult to detect after anisosomes reach a steady state.

Our study supports the notion that TDP-43, at high concentrations, can change from a demixing-prone, liquid state into a gel-like state en route to forming amorphous aggregates ^12, 35^. We found that proteasome and HSP90 inhibition or enhancing XPO-1-mediated nuclear egression promotes the conversion of TDP-43 anisosomes into a gel-like state. By contrast, inhibition of RNA splicing, protein translation, or nuclear export all favors the liquid state of TDP-43.

The roles of RNA splicing and nuclear export in TDP-43 phase regulation might be mechanistically linked because anisosomes stabilized upon nuclear export inhibition are sensitive to RNAase treatment in semi-permeabilized cells. Anisosome maturation probably involves one or more RNA splicing intermediate. Therefore, the completion of RNA splicing followed by nuclear egression of spliced RNAs would disfavor anisosome growth and maturation.

Because the TDP-43 2KQ mutant lacks RNA binding activity, anisosome maturation is likely modulated by other RNA-binding factors. In this regard, TDP-43 is known to interact with many splicing factors including members of the hnRNP family like hnRNP A1/A2 and PSF ^36^; Inhibition of RNA splicing might alter TDP-43’s interactions with these factors, trapping it in an RNA-containing complex that favors anisosome growth and maturation, This could explain why both PlaB and LMB reduce the number of TDP-43 anisosomes while maintaining their dynamic liquid property. Future studies are required to elucidate the precise mechanism by which nuclear transport and RNA splicing modulate anisosome dynamics.

Our validation of proteasome inhibition as a driver of TDP-43 droplet enlargement and hardening reinforces the idea that impaired protein clearance promotes the transition from liquid condensates to gellike or solid aggregates. This is consistent with previous studies in ALS models, which showed that proteasome stress accelerates TDP-43 aggregation and toxicity ^25^. In contrast, inhibition of deubiquitinases reduced the number of TDP-43 anisosomes, probably by promoting the proteasome- mediated clearance of TDP-43. The role of HSP90 in this process may be linked to TDP-43 folding stability, as HSP90 can bind TDP-43 to prevent promiscuous interactions ^37^. Collectively, these observations suggest that TDP-43 condensates are maintained by a delicate balance between formation and clearance, modulated by proteolytic and chaperone activities.

While out chemical and genetic screens have identified multiple pathways influencing TDP-43 phase states, a particularly compelling aspect of our study is the discovery that the nuclear export receptor XPO1 governs TDP-43 liquid-to-solid transitions and subcellular localization. Pharmacological inhibition of XPO1 mitigates cytoplasmic p-TDP-43 accumulation, whereas XPO1 overexpression promotes the cytoplasmic accumulation of TDP-43-containing puncta. However, XPO1 may modulate TDP-43 nuclear egression indirectly, as suggested previously ^32, 38^. While previous studies have not detected strong interactions between XPO-1 and TDP-43 ^32^, a weak or indirect interaction between XPO-1 and TDP-43 may still exist, especially when TDP-43 undergoes demixing. This model would explain why anisosome induction depletes endogenous XPO-1 from the nucleoplasm. Our findings also hint at a potential feedback mechanism in which TDP-43 demixing perturbs nuclear export, analogous to how cytoplasmic TDP-43 aggregates disrupt nuclear import. Given that ALS is characterized by cytoplasmic TDP-43 mislocalization and hyperphosphorylation, the XPO-1-dependent nuclear export pathway may constitute a key molecular switch linking physiological phase behavior to pathological aggregation.

Together, our results support a model in which multiple cellular pathways including proteostasis, RNA metabolism, protein translation, and nuclear export—cooperatively determine the phase behavior of TDP-43. These findings have deepened our understanding of how physiological phase separation can evolve into pathogenic aggregation through cumulative failures of cellular quality control and nuclear export systems.

## Methods

### RNAi-based genetic screen for anisosome regulators

Genome-wide RNA interference (RNAi) screen was performed using DLD1 cells stably expressing Clover-tagged TDP-43 2KQ. Cells were transfected with siRNAs targeting 21,404 genes for 72 hours, followed by induction of anisosome formation with doxycycline (0.5 μg/mL) for 24 h. Specifically, the screen was carried out in 384-well format with 3 individual siRNAs for each gene. To prepare siRNA transfection, control siRNA (40 nmol) was mixed with 2 mL water to create a 50X (20 μM) stock and siRNA-death (20 nmol) was mixed with 1 mL of water for the same concentration. Dilute siRNA stocks to a 400 nM working concentration in water. For each well, mix 0.1 μL transfection reagent in 20 μL of cell culture medium without FBS or P/S. Add 2 μL of siRNA solution to each well and incubate with RNAiMAX for 30 minutes at room temperature. While incubating RNAiMAX, prepare trypsinized cells by ensuring thorough separation and accurate cell counting. Add 20 μL of cell suspension at a density of 0.43 × 10^5^ cells/mL (850 cells/well) in medium containing 20% FBS and 2 x P/S to achieve a final volume of 40 μL per well. Incubate cells for 3 days before addition of 10 μL 10 x doxycycline solution to each well (final volume 50 μL) for anisosome induction. 24 h later, high-throughput (HT) imaging and automated phenotypic screening were conducted to identify genes that modulated anisosome formation. To control for siRNA transfection efficiency, we routinely included a positive knockdown control in which cells were treated with a mixture of siRNAs targeting several essential genes (AllStars Hs Cell Death siRNA (Qiagen, #1027299).

Fluorescence imaging was conducted using an Opera Phenix High-Content Screening System (PerkinElmer). Whole-well images were acquired in confocal mode using a 20X water objective lens after anisosome induction for 24 h. Images were reconstructed and analyzed using the PerkinElmer Columbus server (v2.9.1). Nuclei were segmented based on Hoechst33342 staining, and anisosomes were detected via YFP signals within the nuclear region. The mean anisosome count per nucleus was calculated to assess changes in anisosome levels, while toxicity was evaluated by counting the total number of nuclei. Data were normalized to the siRNA-Neg control for each plate, and Z-scores were calculated to identify significant hits. Identified 1,533 genes whose knockdown significantly reduced the number of anisosomes per cell (Z-score > 2) were subjected to protein network analysis by STRING to identify candidate anisosome regulatory genes that were subject to a second-round screen.

### A chemical genetic screen for anisosome regulators

We also performed a chemical genetic screen using LOPAC^R1280^ (Sigma) compound library on DLD1 cells expressing Clover-tagged TDP-43–2KQ. Briefly, cells were induced with doxycycline for 24 h to promote anisosome formation, followed by treatment with individual compounds for an additional 24 hours. High-throughput (HT) imaging and automated phenotypic screening were conducted to identify compounds that modulated anisosome formation.

### Visualization and analysis of anisosome dynamics

To examine the impact of drug treatment on anisosome size, cells were induced to form anisosome for 24 h and then treated by different inhibitors as follows: cycloheximide (CHX, 20 μg/mL), anisomycin (ANS, 200 nM) for 16 hours. For splicing inhibition, cells were exposed to Pladienolide-B (PlaB) at concentrations of 5 or 20 nM, or DMSO (control) for 16 hours before imaging. Cells were fixed with 4% paraformaldehyde in Phosphate Buffer Saline (PBS). Randomly selected fields were used to quantify the number of anisosomes per cell with at least two biological repeats.

To image anisosome fusion, live cell imaging was performed by treating cells with Leptomycin B (200 nM) or PlaB (20 nM) for 5 hours and then imaged by confocal 3-D sectioning for 30 minutes to capture anisosome fusion events. For Fluorescence Recovery After Photobleaching (FRAP), cells were treated with DMSO, Leptomycin B, Geldanamycin or PlaB as indicated in figure legends. Either part of an anisosome or entire anisosome was photobleached and imaged. TDP-43 fluorescence recovery after photobleaching was quantified by ImageJ and calculated to assess anisosome dynamics. At least 10 anisosomes for each condition were bleached and analyzed.

To evaluate the time course of Leptomycin B (LMB)-induced anisosome changes, DLD1 TDP-43 2KQ-Clover cells were seeded and induced with 0.5 μg/mL doxycycline for 24 hours. Cells were treated with 10 μM LMB and fixed at 0-, 8-, and 16-hours post-treatment. Fixed cells were stained with Hoechst to visualize nuclei. 10-20 confocal 3-D sections were obtained for each randomly selected field. Images were converted to maximum projected view by ImageJ before anisosome counting and intensity measurement. The density plot of anisosome counts per cell was generated using R, illustrating dose-dependent effects of leptomycin on anisosome formation.

To test the effect of XPO1 overexpression on TDP-43 phase regulation, DLD1 TDP-43 2KQ-Clover cells were co-transfected with mCherry-tagged XPO1 (mCh-XPO1) for 24 h before doxycycline was added at 0.5 μg/mL to induce anisosome formation for 24 h before confocal imaging.

To study the relationship between anisosome induction and endogenous XPO1 localization, cells were seeded, induced with 0.5 μM doxycycline for 48 hours, and fixed with paraformaldehyde in PBS. Immunostaining was performed using an XPO1 antibody (Cell Signaling, 46249S, 1:125) and Hoechst nuclear stain. Fluorescence intensity of XPO1 in both induced and uninduced cells was measured by ImageJ.

### Semi-permeabilized cell experiments

To reconstitute anisome disassembly and re-assembly in seminar permeabilized cells, 30,000 DLD1 cells were seeded in an eight-well ibidi cell chamber two days before the experiment. Cells were treated with Doxycycline for 24 h to induce anisosomes and then treated with inhibitors as indicated in the figure legends. We then washed Cells three times with ice-cold PBS containing 2 mM MgCl2 (PBS-Mg) and then treated these cells in the same buffer (300 μl) containing 200 units of SLO (Sigma-Aldrich, cat. no. SAE0089) on ice for 30 min. The cells were then washed two times with PBS-Mg buffer and incubated with 200 μl of reagent mixture containing 200 μl of PBS in the absence of presence of ARS/GTP or 200 μl of cow liver cytosol (CLC) in the presence of the NucSpot dye. Cells were either imaged directly or fixed with 4% paraformaldehyde before imaging by a Nikon CSU-W1 SoRa super-resolution confocal microscope.

### Experiments with organoids

Stem cell gene editing of the patient K181E mutation in iPSC cells was reported previously ^16^. Differentiation of iPSCs into forebrain neurons was performed following a previously published protocol ^39^. Briefly, the procedure used an induction medium (IM-N2) and a neuronal culture medium (CM). IM-N2 was prepared with Knockout DMEM/F12, N2 supplement, NEAA, Gluta-MAX, Chroman I, and doxycycline. On Day 0, iPSCs were observed for confluency, dissociated with Accutase, centrifuged, and resuspended in IM-N2 with Chroman I, and then seeded into Matrigel-coated plates. Over the next four days, cells were monitored microscopically for neurite extensions while media containing doxycycline is refreshed daily. By Day 4, cells exhibit neurite growth and are ready for replating onto poly-L-ornithine (PLO)-coated dishes, prepared in advance by coating with PLO solution, incubating, washing, and drying. Replated cells were cultured in CM comprising BrainPhys medium, B27+ supplement, neurotrophic factors (GDNF, BDNF, NT-3), laminin, and doxycycline, to promote neuronal maturation.

3-D culture and organoid growth were performed as previously described ^40^. In brief, iPSCs grown on Matrigel were dissociated into single cell suspension by Versene solution (ThermoFisher) and seeded into a 12-well Aggrewell plate (Stemcell Technologies) at 4,000 cells/ well. Next day, spheroids were transferred to an ultralow attachment plate (Corning) containing phase I medium: DMEM/F12, 20% Gibco KnockOut Serum Replacement, 1X Glutamax, 1X MEM Nonessential Amino Acid, 55 μM β-Mercaptoethanol, 1X Pen/Strep, 2 μM Dorsomorphine, 2 μM A83-01. After 5 days, medium was switched to phase II medium: DMEM/F12, 1X N-2 Supplement, 1X Glutamax, 1X MEM Nonessential Amino Acid solution, 1X Pen/Strep, 4 ng/mL WNT3a, 1 μM CHIR-99021, 1 μM SB-431542. On day 7, spheroids were embedded into Matrigel and allowed to continue growing for 7 more days. On day 14, individual spheroids were manually freed from the Matrigel and transferred to a SpinOmega bioreactor spinning at 120 RMP with phase III media: DMEM/F12, 1X N-2 Supplement, 1X B-27 Supplement, 1X Glutamax, 1X MEM Nonessential Amino Acid, 55 μM 2-Mercaptoethanol, 1X Pen/Strep, 2.5 μg/mL insulin. 50 days later, the medium was switched to final differentiation medium: Neurobasal medium, 1X B-27 Supplement, 1X Glutamax, 55 μM 2-Mercaptoethanol, 1X Pen/Strep, 0.2 mM Ascorbic acid, 0.5 mM cAMP, 20 ng/mL brain-derived neurotrophic factor (BDNF), 20 ng/mL glial-derived neurotrophic factor (GDNF). 107-day-old organoids were dissociated into single cells using a 50:50 mixed of Accutase and 0.25% Trypsin with DNase I (1 mg/mL) and plated onto chambered glass slides pretreated with 1% Matrigel (Corning, Inc). Alternatively, organoids were fixed and sectioned for immunostaining (see below). For drug treatment, forebrain organoids (87 Day) were transferred to a 12-well plate on an orbital shaker (120 RPM) and treated with 20 nM KPT276 in final differentiation medium. Medium was changed every other day (Dexoregen, Inc). The organoids were harvested and frozen after 35 days of treatment for sectioning and immunostaining. Each experiment was performed with two batches of organoids per condition to reduce the effect of batch variation. The wildtype cells and K181E mutant have the same genetic background.

### Tissue preparation and immunostaining

Organoids were fixed in 4% paraformaldehyde in PBS for 1 hour at room temperature, washed with PBS, and immersed in 15% sucrose/PBS solution overnight. Subsequently, organoids were embedded in O.C.T. compound (Sakura) in a plastic mold and frozen down in an ultralow freezer. Embedded organoids were sliced onto glass slides using a Cryostat (Leica). Slides were rinsed with PBS, permeabilized with 0.5% Triton-X/PBS solution for 1 hour at room temperature and blocked using 1% donkey serum in 0.1% Tween-20/PBS solution for 1 hour. Primary antibodies, chicken anti-TUJ1 (Abcam, Ab41489, 1:1,000 dilution), rabbit anti-TDP-43 (10782-2-AP, 1/1000 dilution), mouse anti-phospho-TDP-43 (Cosmobio, CAC-TIP-PTD-M01A, 1/500 dilution), were added to the slides and the slides were incubated in a humidified chamber at 4 °C overnight. After several washes in 0.1% Tween-20/PBS solution, DAPI (Sigma) or secondary antibodies, donkey anti-rabbit, anti-mouse, and anti-chicken (Jackson ImmunoResearch), diluted 1:1,000 in blocking solution, were added to the slides. After 1 hour of incubation at room temperature, the slides were washed and mounted in antifade mounting solution (Fisher scientific).

### Image acquisition and processing

Fluorescence confocal z-section images were acquired using a Nikon CSU-W1 SoRa microscope equipped with temperature and CO_2_ control enclosures and a 60x TIRF lens. Randomly selected fields were imaged each with ~25 slices. Maximum intensity projection views were reconstructed and analyzed by the open-source Image J software (also named Fiji). 3-D and time lapse visualizations were achieved by the Imaris software. Fluorescence intensity was quantified using Fiji. To automatedly count anisosomes, maximum intensity projection views were split into individual channels by Fiji. A consistent default threshold method was applied to each channel. The particle analysis function was used to automatically identify anisosomes or other fluorescence structures for size and intensity measurement. Statistical analyses were conducted using Excel (for Student’s t-test) or GraphPad Prism versions 8.0, 9.0, 11. P-values were calculated using Student’s t-test in Excel or one-way ANOVA in GraphPad Prism. Curve fitting, including linear and nonlinear models, as well as IC_50_ calculations, were also performed using GraphPad Prism versions 8.0, 9.0 and 11.

### Statistic and reproducibility

All statistical analyses were conducted with GraphPad Prism v10 or Excel. Statistical methods and the number of cells or brain organoid samples (N) are indicated in figure legends or shown in figures as individual data point. Biological repeats (n) are specified in figure legends. No biological repeat was excluded from the analyses. All experiments were repeated at least twice with individual data point labeled in figures unless specified. For imaging analyses, cells in randomly selected fields were analyzed. The researchers were not blinded. The iPSC cell line was derived from a ADRDs genetic risk-free clone, which was initially obtained from a male. Figures were prepared using ImageJ 1.54f, Imaris 9.9.0, Adobe Photoshop v25.12.1, and Adobe Illustrator 28.7.4.

## Supplementary Material

Supplement 1

## Figures and Tables

**Figure 4:** Anisosome phase behavior is modulated by RNA splicing and protein translation **(A)** The splicing inhibitor Pladienolide-B (PlaB) reduces anisosome number in a dose dependent manner. Representative images of anisosome-induced cells treated with DMSO (control), 5 nM, or 20 nM PlaB for 16 h. Shown are maximum intensity projection views. Scale bar: 10 μm. **(B)** Quantification showing the number of anisosome (AS) per cell in TDP-43 2KQ-Clover cells treated with PlaB as indicated. * p <0.05, ** p <0.01, **** p < 0.0001 by ordinary one-way ANOVA and Dunnett’s multiple comparisons. N=3 biological repeats, and in each experiment, at least 30 randomly selected cells were analyzed. **(C)** Representative z-section confocal FRAP images of anisosomes in cells treated for 16 h with DMSO or PlaB (20 nM). BB, before photobleaching, right after photobleaching (0 s), or 4 and 30 seconds after photobleaching (4 s and 30 s). Scale bar, 1 μm. **(D)** The graph shows the quantification of the remaining TDP-43 fluorescence (FL) in C. Error bars indicate mean ± SD, N = 28 for control and 23 for PlaB-treated cells. MFI, Mean Fluorescence Instensity. **(E)** Live cell imaging of anisosome fusion in TDP-43 2KQ-Clover cells treated with 20 nM PlaB for 5 h before tracking the fusion. Representative reconstructed 3-D images from Movie S1 showing fusion events indicated by arrows. Scale bar, 1 μm. **(F)** Representative maximum intensity projection views of anisosome-induced (24 h) cells treated with DMSO (control) or ANS (200 nM) for 16 h. Scale bar, 1 μm. **(G)** Quantification of the number of anisosomes per cell in randomly selected images of DMSO-, Cycloheximide (CHX)-, or ANS-treated cells. **** p < 0.0001 by ordinary one-way ANOVA and Dunnett’s multiple comparisons. Each dot represents a randomly selected field with at least 20 cells counted from one of the 3 biological repeats. **(H)** Quantification of anisosome size in control or cells treated with CHX or ANS. ****, p < 0.0001 by ordinary one-way ANOVA and Dunnett’s multiple comparisons. N=3 biological repeats. AU, arbitrary unit. **(I)** As in **C** except that anisosome-induced cells were treated with CHX or ANS before photobleaching. Scale bar 1 μm. **(J)** Quantification of fluorescence recovery in CHX- or ANS-treated cells vs the DMSO control. N=10 anisosomes/condition.
